# Localized Pigmented Villonodular Synovitis of the Posterior Knee Compartment with Popliteal Vessel Compression: A Case Report of Arthroscopic Resection Using Only Anterior Knee Portals

**DOI:** 10.1155/2018/7532358

**Published:** 2018-06-21

**Authors:** Jack Daoud, Dany Aouad, Youssef Hassan, Georges El Rassi

**Affiliations:** Department of Orthopedic Surgery and Traumatology, Saint Georges University Medical Center, Balamand University, P.O. Box 166378, Achrafieh, Beirut 1100 2807, Lebanon

## Abstract

**Background:**

Pigmented villonodular synovitis is a rare pathology causing hyperplasia of the synovium. It mostly affects young populations and most commonly the knee joint. It rarely affects the posterior compartment of the knee as the case presented in this study. Open surgery is usually used to treat this condition; however, in our case it was excised arthroscopically despite the anatomical challenges of the posterior knee compartment.

**Case Presentation:**

This case presents a female patient with a complaint of posterior-region pain of her left knee post direct trauma post fall. This was directly followed by knee joint blockage for 1-week duration before presentation to the hospital. On MRI, she was found to have a multiloculated hemosiderin-containing structure of synovial origin within the femoral notch, extending beyond the joint capsule displacing the popliteal vessels. The patient underwent arthroscopic resection of the lesion, which was found to be pigmented villonodular synovitis on anatomopathological examination. On 6-month follow-up, the patient showed good clinical evolution with the absence of symptoms and back-to-normal daily activities.

**Conclusion:**

This is a rare case of PVNS affecting the posterior knee joint compartment of a middle-aged woman, which was successfully excised arthroscopically, with no residual affected tissue or recurrence on 6-month follow-up.

## 1. Introduction

Pigmented villonodular synovitis (PVNS) is characterized by hyperplasia of the synovium or tendon sheath. In 80% of the cases, it affects the knee joint [1].

It is mostly seen among patients between 20 and 50 years of age, with a slightly higher occurrence among females [2].

The cause of PVNS is unknown; however, repetitive trauma, intra-articular bleeding, and inflammation play a major role. It presents with cytogenetic abnormalities and its capacity for autonomous growth suggests that it has a neoplastic nature [3, 4].

Patients usually present with progressive symptoms which include pain, repeated nontraumatic joint effusion, and decreased range of motion in the diffuse form. In the localized form, the symptoms may mimic meniscal lesions with problems of abnormal mobility [5, 6].

The treatment of PVNS requires the resection of the lesion by arthroscopy or through open excision, with local recurrence of 18 to 46% [5, 7].

## 2. Case Report

A 49-year-old previously healthy female presented with a 1-week posterior-region knee pain post direct fall from a standing position on her left knee. This was directly followed by knee joint blockage with a limited range of motion of only 30 to 60 degrees of flexion. The patient started taking NSAID and had minimal pain relief. She reported increased pain upon standing from a sitting position and vice versa with associated tingling and numbness at the level of the calf region especially upon standing.

On examination, she showed good lower-limb alignment, no pain was provoked on meniscal and ligament testing, and there was an absence of muscular atrophy. Her range of motion was limited to only 30 to 60 degrees of flexion, and the patient had pain on the active range of motion. She also had pain upon active and passive extension of the knee joint with tingling and numbness over the calf region extending from the knee posteriorly. She was found to have a nonpitting edema posteriorly with moderate anterior joint effusion. On patellar examination, she felt pain originating from the posterior region of her left knee joint. To note, the patient has never had any symptoms related to her knee up until the direct fall from the standing position.

**Figure 1:**
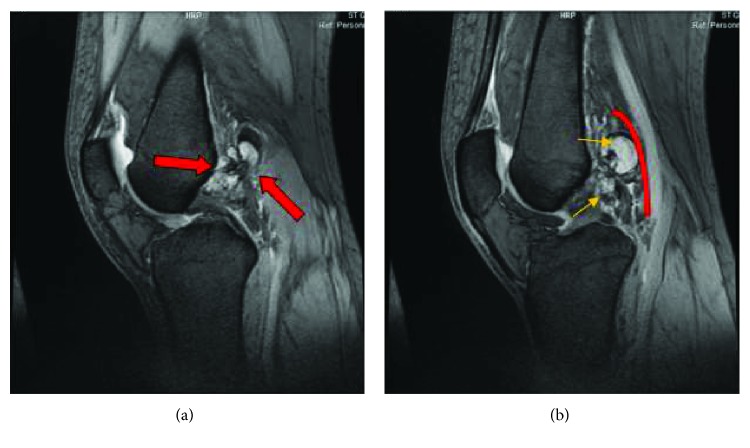
(a) Lateral-view T2 sequence MRI of the left knee showing a multiloculated structure arising from the synovium around the cruciate ligaments within the femoral notch. (b) The lesion is extending beyond the joint capsule posteriorly displacing the popliteal vessels.

MRI revealed a multiloculated structure arising from the synovium around the cruciate ligaments within the femoral notch extending beyond the joint capsule posteriorly with significant displacement of the popliteal vessels (Figures 1(a) and 1(b)).

**Figure 2:**
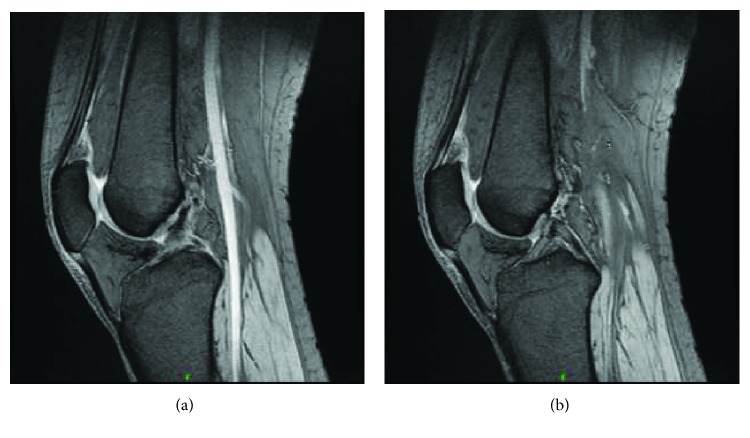
(a) Lateral-view T2 sequence MRI of the left knee 6-month post arthroscopic resection of posterior-compartment PVNS, showing no more compressive effect on both the cruciate ligaments and the popliteal vessels. (b) There is a significant decrease in the soft tissue enhancement suggestive of decreased inflammation in the region of the resected lesion. There is no evidence of interval appearance of new susceptibility artifacts or blooming effect suggesting recurrence.

It showed evidence of synovial thickening, and on gradient echography, it showed spotty and irregular hyposignals compatible with the presence of hemosiderin. There also was associated soft tissue edema around the above-described lesion. The patient underwent arthroscopic intervention in the left knee under spinal anesthesia. The posterior compartment of the knee was reached arthroscopically through the triangular space formed by the ACL laterally, PCL medially, and the femoral notch superiorly. Total resection of the lesion was done through only anterior knee portals without taking the risk of posterior portals preventing potential neurovascular injury. The pathology report confirmed the presence of PVNS. On 6-month MRI follow-up, the previously described soft tissue enhancement suggestive of residual inflammatory changes noted within the femoral notch along the cruciate ligaments as well as posterior to the distal metaphysis at the site of the previously present lesion has decreased (Figures 2(a) and 2(b)). The synovial fluid had also decreased with no evidence of hemosiderin deposits. There was no evidence of interval appearance of new susceptibility artifacts or blooming effect on the T2 gradient sequence that could correspond to evident recurrence. The compressive effect previously present on the popliteal vessels has completely disappeared with no further tingling sensation and numbness over the calf region. Clinically, patient regained full ROM on flexion extension (0° to 135°) of the knee. The previously reported pain no longer exists, with no blocking upon flexion. Patient underwent physical therapy protocol and showed significant muscle strength and balancing improvement. She was scheduled to 1-year clinical follow-up.

## 3. Discussion

Pigmented villonodular synovitis is an uncommon disease with around two new cases per million individuals per year [1]. It has a benign proliferative nature and affects the synovium of a joint either diffusely or focally [8].

Characterized by hyperplasia of the synovium along with hemosiderin deposition and multinucleated giant cells, PVNS affects the knee joint in 80% of the cases [1]. Young people between the ages of 20 and 50 years show more prevalence to PVNS than other age groups. Patients usually present with vague symptoms like pain in most cases, swelling, and decreased range of motion.

In most cases, these symptoms are chronic in nature. However, this does not rule out an acute onset of symptoms such as the above-presented case.

Clinical presentation is not diagnostic in most cases, therefore imaging studies are needed. Ultrasonogrophy helps in determining the nature of the mass, whether liquid or solid, but is not diagnostic and specific. MRI has characteristic features distinguishing PVNS, mainly the presence of hemosiderin deposition, especially through gradient-echo pulse. The latter also helps in checking the involvement of the disease in concordance with its anatomical surrounding such as tendon sheath, ligaments and so on [3].

Treatment modalities are in ongoing research and studies. Total resection of the mass lesion of affected synovium is still the gold standard. Adjuvant medications are being studied with the aim of decreasing the rate of recurrence, however this is beyond the scope of this article.

In the above case, the posterior-compartment lesion was found to be in contact with the popliteal vessels and was affecting the cruciate ligaments anteriorly. Open excision was not performed due to the invasiveness of the surgery and the location of the lesion in the posterior compartment which would necessitate extensive dissection. Knee arthroscopy was used with successful total resection of the lesion. This was done using only anterior knee portals, decreasing the risk of possible neurovascular injuries if posterior portals were introduced. In order to reach the posterior femoral condyle and ensure total resection, both the electrocautary and the shaver were manually bent successfully reaching the affected area uniquely using anterior portals. This was followed by a 6-month follow-up MRI, showing a complete resection with no signs of recurrence along with remarkable clinical improvement on physical examination. In the literature, Keyhani S. et al. [9] did a case study consisting of 21 patients having diffuse pigmented villonodular synovitis of the knee who underwent total arthroscopic resection, using posteromedial, posterolateral, anteromedial, and anterolateral knee portals. On follow-up, no recurrences were found with no residual symptoms. In addition, another article presenting the surgical outcomes of 26 patients with diffuse knee PVNS were studied on 4-year follow-up [10]. They found that staged open total synovectomy with a posterior and then an anterior approach seems to be a superior method for surgical treatment of diffused forms. The above-described case is one of the few arthroscopically removed localized PVNS found in the vicinity to the popliteal vessels, using only anterior portals with manual bending of both the shaver and electrocautery in order to optimally reach the posterior affected compartment, without the use of any posterior knee portals, aiming to prevent any neurovascular injury and taking into consideration the compressive effects the PVNS had on the popliteal vessels.

## 4. Conclusion

Pigmented villonodular synovitis is a benign, neoplastic, rare pathology most commonly affecting the knee joint. Patients clinically present with nonspecific symptoms, and it can be confused with other more common pathologies like meniscal tears, and so on. Upon suspicion, imaging is required to guide towards diagnosis. MRI imaging usually shows mass-like proliferation of the synovium with lobulated margins, either diffuse or localized, along with low-intensity signals due to haemosiderin deposition. Treatment is usually done by excision of the lesion either with open surgery or arthroscopically, depending on location, type, surrounding structure involvement, and invasiveness. Despite total resection with acceptable margins, there is a risk of recurrence of about 40%.
